# 

**Published:** 1836-01

**Authors:** 


### QUARTERLY JOURNAL

OF

### PRACTICAL MEDICINE AND SURGERY

STATE OF LOUISIANA CHARITY HOSPITAL * MEDICAL LIBRARY * JUSTICE

LIBRARY OF THE

Orleans Parish Medical Society

EDITED BY

JOHN FORBES M.D. F.R.S.

AND

JOHN CONOLLY M.D.

EDITORS OF THE CYCLOPÆDIA OF PRACTICAL MEDICINE

VOL. I

JANUARY—APRIL 1836

LONDON
SHERWOOD GILBERT AND PIPER
PATERNOSTER ROW
MDCCCXXXVI

LONDON:
PRINTED BY J. AND C. ADLARD, BARTHOLOMEW CLOSE.

### OBITUARY.

THE Profession, in this country, has recently sustained the loss of some distinguished members, of whom we hope to give some account in our next Number. We can at present only record their deaths.

At London, November, 1835, Sir DAVID BARRY, M.D., in his 58th year.

At Edinburgh, on the 27th October, JAMES HAMILTON, M.D., aged 88, the well-known author of the work on Purgative Medicines.

At Edinburgh, November, 1835, J. W. TURNER, Esq., Professor of Surgery in the University of Edinburgh.

At his seat near Winchester, on the 2d December, 1835, PELHAM WARREN, M.D., of London, Fellow of the Royal College of Physicians, aged 57.

### LIST OF ORIGINAL PAPERS PUBLISHED IN THE BRITISH JOURNALS DURING THE LAST QUARTER.*

### THE EDINBURGH MEDICAL AND SURGICAL JOURNAL.

*No.* 125, *Oct.* 1835.

1. Select Cases and Communications, forming Part of the Transactions of the Medico-Chirurgical Society of Edinburgh. Communicated by the Council. [All single cases, except the two following.]
2. Cases of fatal Jaundice, in which the Bile Ducts appeared on Dissection to be pervious and empty; with Observations. By Professor Alison.
3. Abstract of Cases in which a Portion of the Cylinder of the Intestinal Canal, comprising all its Coats, had been discharged by Stool, without the Continuity of the Canal being destroyed. By William Thomson, M.D.
4. On Malposition of the Ovaria, Bladder, and Urethra. By J. T. Ingleby.
5. Observations on the Treatment of Fistula Lachrymalis. By E. Lubbock, M.D.
6. Contributions to the Statistics of the British Army. By Henry Marshall.
7. Observations on Petechial Fevers and Eruptions. By J. H. Peebles, M.D.
8. On Diseases simulating acute inflammatory Attacks of various important Organs, and dependent on Ganglionic and Spinal Irritation. By John Torbet, Esq.

### THE DUBLIN MEDICAL JOURNAL.

*No. XXII. Sept.* 1835.

1. Case in which several Metallic Bodies were found in the Stomach. By Robert Harrison, M.D. &c.
2. Report of a singular Case of Fracture of the Pelvis. By John Houston, M.D.
3. Observations on Diseases of the Stomach, their Sympathies and Complications. By Langston Parker, M.R.C.S.L.
4. First Report of the New Lying-In Hospital, Dublin, for 1834. By T. E. Beatty, M.D. Master of the Hospital, &c.

*No. XXIII. Nov.* 1835.

5. Practical Observations in Midwifery. By W. F. Montgomery, M.D.
6. Cases of Aneurism, in which some unusual Circumstances were observed. By John Brown, M.D.
7. An Account of Hydatids in the Omentum of Deer, with Observations on their Pathological Changes. By J. Houston, M.D.
8. Case of Poisoning by Sulphuric Acid, with Remarks. By W. Corbet, M.B.
9. Remarks on Partial Amputation of the Foot. By Francis Rynd, A.M. &c.
10. On the Structure of the Mammary Gland in the Cetaceæ, &c. By Arthur Jacob, M.D.
11. On Venereal Diseases of the Testicle. By J. W. Cusack, Esq.
12. A Case of Poisoning by Hydrocyanic Acid, with Observations. By G. T. Geoghegan, M.D. &c.
13. Case of Pulsation of the Veins of the Upper Extremities, with Observations. By Charles Benson, M.D. &c.
14. On the Treatment of Croup. By Dr. Kirby.

### THE LANCET.

*No.* 627, *Sept.* 5, 1835.

1. On the external Use of Opium in the Treatment of Bronchitis and Croup. By Dr. Bow.
2. Report of the Cork-street Fever Hospital, Dublin. By John Eustace, M.D.

*No.* 630, *Sept.* 26.

3. Observations on the Nature of Inflammation and Irritation. By Henry Searle, Esq.

*No.* 633, *Oct.* 17.

4. Reflections on Infantile Remittent Fever. By John Alexander, M.D.
5. On the Use of the Supersulphate and Supertartrate of Iron in several Diseases. By J. P. Buckland, Esq.

*No.* 634, *Oct.* 24.

6. On the Nature of Inflammatory Fever. By H. Searle, Esq.
7. On an Instrument for Auscultation in Vesical Calculi. By Claudius Tarral, M.D.

*No.* 635, *Oct.* 31.

8. On the injurious Effects of Splints and Tight Bandaging in Fractures of the Bones. By W. C. Radley, Esq.

*No.* 638, *Nov.* 21.

9. On the Treatment of Fractures without the Aid of Splints or Tight Bandages. By W. C. Radley, Esq.

* In this list we have omitted the Reports of Lectures, Hospital Reports, and, in general, histories of single cases; also controversial discussions and other matters of temporary interest.

10. Reflections on Infantile Remittent Fever. By John Alexander, M.D.

*No.* 640, *Dec.* 5.

11. On the Nature and Qualities of Flame. By W. R. Clanny, M.D.

12. On the Law of Mortality in each County of England. By R. T. Edmonds, Esq.

13. Case of Gangrena Senilis, with Remarks. By W. Tagert, Esq.

*No.* 641, *Dec.* 12.

14. Of Malignant Disease of the Testis. By George Langstaff, Esq. Surgeon.

---

THE LONDON MEDICAL GAZETTE.

*No.* 405, *Sept.* 5, 1835.

1. Contributions to the Diagnosis and Pathology of Thoracic Diseases. By Charles Cowan, M.D.

2. Observations on the Changes produced in the Blood in the Course of its Circulation. By C. J. B. Williams, M.D. &c.

3. Objections to Dr. Reid's Experiments on the Nervous System and Muscular Irritability. By J. W. Earle, Esq.

4. Does Arsenic produce Ptyalism? The Affirmative, illustrated by Cases. By Edward Furley, Esq.

*No.* 406, *Sept.* 12.

5. Remarks on Dr. Hope's Conclusions from certain Experiments on the Sounds of the Heart. By C. J. B. Williams, M.D. F.R.S.

6. Midwifery Hospital Reports for 1834. By E. Rigby, M.D. F.L.S.

*No.* 408, *Sept.* 26.

7. Report of Surgical Cases treated in the Liverpool Northern Hospital. By J. M. Banner, Esq.

8. On the complicated Forms of Venereal Diseases. By D. H. Walne, Esq.

*No.* 409, *Oct.* 3.

9. Notice of Dr. E. Harrison's Mode of distinguishing the Boundaries of the Lungs, Liver, &c. By Dr. C. J. B. Williams.

10. On the Functions of the Lingual Branches of the Fifth and Eighth Nerves. By H. Mayo, Esq. F.R.S. &c.

*No.* 410, *Oct.* 10.

11. On the Cholera of North America. By G. Farr, Esq.

*No.* 411, *Oct.* 17.

12. On the Application of Comparative Anatomy to Human Embryology. By John Anderson, M.G.S.

13. Observations on a fatal Case of Confluent Small Pox. By S. J. Jeaffreson, M.B.

14. On Fracture of the Patella, with a Description of a new Instrument. By E. F. Lonsdale, Esq.

15. Observations on Animal Temperature. By A. P. W. Philip, M.D.

*No.* 412, *Oct.* 24.

16. On the Climate of Madeira, and its true Value to the Consumptive. By J. M. Calvert, M.D.

*No.* 413, *Oct.* 31.

17. On the Efficacy of Ice in Dyspeptic Disorders. By F. Bailey, M.D.

18. On M. Baudelocque's Improvements in Practical Midwifery. By J. C. Cooke, Esq.

19. Report of Fractures treated in the London Hospital. By John Adams, Esq.

*No.* 414, *Nov.* 7.

20. Some Observations on Animal Temperature. By A. P. W. Philip, M.D.

*No.* 415, *Nov.* 14.

21. Practical Suggestions for the Improvement of the next Census. By John Rickman, Esq.

22. On Paralysis in Children. By John Badham, M.D.

*No.* 416, *Nov.* 21.

23. On the Principles regarding Life and Mind. By John Roberton, Esq.

24. On the Efficacy of Sea Water as an internal Remedy. By Ed. Greenhow, M.D.

25. Experiments on the Nerves of the Tongue, proving that the Function of Taste is distinct from that of common Sensation. By Daniel Noble, Esq.

26. On Discharge of Pus from the Ear, after Injuries of the Head. By Cæsar Hawkins, Esq.

27. On the Cure of Prolapsus Uteri. By G. O. Heming, M.D.

*No.* 418, *Dec.* 5.

28. Report of the Diseases of St. Mary-le-bone. By J. Clendinning, M.D.

29. Structure and Uses of the Intervertebral Substance. By H. Labatt, A.B.

30. Report of Fractures treated in the London Hospital. By John Adams, Esq.

31. On the Nervous Influence and Animal Heat. By C. J. B. Williams, M.D.

32. On the small Size of the Pelvis in Rickets. By A. Shaw, Esq.

*No.* 419, *Dec.* 12.

33. On certain Theories regarding the Sounds of the Heart. By R. H. Meade, Esq.

34. On the Trichina Spiralis, the Animal found in Human Muscle. By Arthur Farre, M.B.

35. On the Physiology of the Fifth Pair of Nerves. By John Bishop, Esq.

36. Sequel of the extraordinary Case of Abstinence at Ayr. By C. F. Sloan, M.D.

37. On Starvation in a Coal Mine. By Charles Thornhill, Esq.

38. Observations and Researches on a New Method of Curing Cancer. By Alex. Ure, M.D. M.R.C.S.

39. Some Remarks on the Nervous System of Insects. By John Anderson, Esq.

40. On the Nature of the Nervous Influence. By A. P. W. Philip, M.D.

41. On the Faulty and Defective System of our Hospitals in respect to Nurses and Sisters. By John Chatto, Esq.

42. Efficacy of Iodine in Secondary Syphilis. By Forbes Winslow, Esq. M.R.C.S.L.

---

### THE LONDON MEDICAL AND SURGICAL JOURNAL.

*No.* 195, *Oct.* 24.

1. On the Diseases of the Natives on the Banks of the River Niger. By K. A. K. Oldfield, Esq.

*No.* 196, *Oct.* 31.

2. A Sketch of the Medical and Statistical History of Epidemic Diseases in Ireland from 1798 to 1835. By W. Stoker, M.D.

3. On the Employment of Capsicum externally in various Diseases. By A. Turnbull, M.D.

*No.* 197, *Nov.* 7.

4. Practical Observations on Midwifery: on Relaxation of the Uterine Tissue. By W. F. Montgomery, M.D.

*No.* 199, *Nov.* 21.

5. Some Practical and Medical Contributions to the Science of Mineral Magnetism. By Dr. Charles Schmidt.

*No.* 202, *Dec.* 12.

6. On Suppuration.—A Paper read before the Medical Society of the London University. By T. Morton, Esq. M.R.C.S.

---

### LIST OF BOOKS RECEIVED FOR REVIEW.

1. A Theoretical and Practical Treatise on Diseases of the Skin. By S. Rayer, M.D. Second Edition. Translated from the French. London, 8vo. pp. 1300, with an Atlas of twenty-six quarto plates. 4l. 8s.

2. A Treatise on the more Obscure Affections of the Brain, on which the Nature and successful Treatment of many Chronic Diseases depend; being the Gulstonian Lectures delivered at the College of Physicians in May 1835. By A. P. W. Philip, M.D. F.R.S.L.E. London, 12mo. 4s.

3. A Treatise on Pulmonary Consumption, comprehending an Inquiry into the Nature, Causes, Prevention, and Treatment of Tuberculous and Scrofulous Diseases in general. By James Clark, M.D. F.R.S. Lond. 8vo. 1835. 12s.

4. Observations on the Climate, Soil, and Productions of British Guiana; with incidental Remarks on the Diseases, their Treatment and Prevention. By John Hancock, M.D. London, 1835. 8vo. p. 89.

5. The Clinique Médicale; or Reports of Medical Cases. By G. Andral, Professor, &c. Condensed and translated, with Observations, &c., by D. Spillan, M.D. Parts I. II. III. 5s. each. *Lond.* 8vo. 1835.

6. An Essay on the Nature of Diseases. By A. Green, LL.B. London 1835. 12mo. 2s.

7. A Statistical Inquiry into the State of the Medical Charities of Ireland; with Suggestions for a Medical Poor Law, &c. By Denis Phelan, Surgeon to the Tipperary Jail, &c. Dublin, 8vo. 1835. 10s. 6d.

8. A Treatise on Hydrocephalus, with the most successful Modes of Treatment. By W. Griffith, &c. London, 1833. 12mo. 3s. 6d.

9. A Treatise on Diet and Regimen. By W. H. Robertson, M.D. London, 1835. 12mo. 6s.

10. Elements of Bedside Medicine and General Pathology, &c. By J. S. Thorburn, M.D. Lond. 1835. 8vo. 14s.

11. Observations on the principal Medical Institutions and Practice of France, Italy, and Germany; with Notices of the Universities, and Cases from Hospital Practice. By E. Lee, M.R.C.S. Lond. 1835. 8vo. 8s.

12. Tables for the Chemical Analysis of Inorganic Bodies. Edin. 1832. 4to. 2s. 6d.

13. On Dropsies connected with suppressed Perspiration and coagulable Urine. By Jon. Osborne, M.D. Lond. 1835. sm. 8vo. 5s.

14. Practical Anatomy of the Nerves and Vessels supplying the Head, Neck, and Chest, intended as a Guide for Students. By E. Cock, Demonstrator of Anatomy at Guy's Hospital, Lond. 1835. 12mo. 7s.

15. Practical Observations on Diseases of the Heart, Lungs, Stomach, Liver, &c., occasioned by Spinal Irritation: and on the Nervous System in general, as a Source of Organic Disease. By John Marshall, M.D. London, 1835. 8vo. 6s. 6d.

16. On Bloodletting. An Account of the Curative Effects of the Obstruction of Blood; with the Rules for employing both local and general Bloodletting in the Treatment of Diseases. By James Wardrop, M.D. Surgeon to the late King, &c. London, 1835. sm. 8vo. 4s.

17. Compendium of the Ligaments; illustrated by Wood-cuts. By A. M'Nab, jun. Member of the Royal College of Surgeons, London. 1835. 12mo. 3s. 6d.

18. Reflections on the Nature of Inflammation, and its alleged Consequences. By David Badham, M.D. one of Dr. Radcliffe's Travelling Fellows from the University of Oxford. Glasgow, 1834. 8vo. pp. 67.

19. The Cyclopædia of Anatomy and Physiology. Edited by R. B. Todd, M.B. Parts I. II. III. IV. London, 1835, royal 8vo. 5s. each part.

20. The Anatomy of the Regions interested in the Surgical Operations performed upon the Human Body, with occasional views of the Pathological Conditions which render the interference of the Surgeon necessary. In a series of Plates, the size of Life. By J. Lebaudy, M.D. Parts I. II. III. London, 1835, folio, 24s.

21. The Nature of Cholera investigated. By J. G. French, M.R.C.S., &c. London, 1835, 8vo. pp. 54.

22. Illustrations of the Botany and other Branches of the Natural History of the Himalaya Mountains. By J. F. Royle, F.L.S. Part VIII. Lond. 1835. 21s.

23. An Essay on Clinical Instruction. By E. C. A. Louis, M.D., &c. Translated by Peter Martin, M.D., M.R.C.S. London, 1835, 8vo. 2s.

24. The Cyclopædia of Practical Medicine. Edited by John Forbes, M.D., F.R.S. Alexander Tweedie, M.D., and John Conolly, M.D. 4 vols. London, 1832-35, royal 8vo. 6l. 15s.

25. The American Cyclopædia of Practical Medicine and Surgery, a Digest of Medical Literature. Edited by Isaac Hays, M.D. Parts I.-VII. Philadelphia, 1833-35, 8vo. 50 cents each part.

26. An Introduction to the Study of Practical Medicine. By J. Macrobin, M.D. 5s.

27. A Practical Treatise on Diseases of the Teeth. By W. Robertson, 1835, 8vo.

28. The Pathology and Diagnosis of Diseases of the Chest; illustrated especially by a rational exposition of their Physical Signs. With new researches on Sounds of the Heart. By C. J. B. Williams, M.D., F.R.S., &c. Third Edition, much enlarged. London, 1835, 8vo.

29. Pathological Researches on Phthisis. By E. C. A. Louis, M.D., &c. Translated from the French, with introduction, notes, additions, and an Essay on Treatment. By Charles Cowan, M.D. Lond. 1835, 12s.

30. A Treatise on Insanity, and other Disorders affecting the Mind. By J. C. Prichard, M.D., F.R.S. Lond, 1835, 8vo. 14s.

31. Pathological Anatomy. Illustrations of the Elementary Forms of Disease. By Robert Carswell, M.D. Professor of Pathological Anatomy in the University of London. Fasciculus I.-VIII. London, 1834-5, folio, 15s. each fasc.

32. A practical Compendium of the Diseases of the Skin. By J. Green, M.D. London, 1835. 8vo. 12s.

33. A Dictionary of Terms used in Medicine and the Collateral Sciences, &c. By R. D. Hoblyn, A.M. London, 1835, small 8vo. 9s.

34. The Medical Student's Guide to the Translation and Composition of Latin Prescriptions. By T. W. Underwood. London, 1835. 12mo.

36. St. Thomas's Hospital Reports. By John F. South, Assistant Surgeon. No. I. Nov. 1835, pp. 118, 3s.

35. A Manual of Aphorisms in Chemistry and Toxicology. For the use of Students preparing for Apothecaries' Hall. By R. Venables, A.M., M.B., Oxon. London, 1836, 7s. 12mo. pp. 270.

36. The Practice in the Liverpool Ophthalmic Infirmary for the year 1834. By Hugh Neill, Surgeon to the Charity. London, 1835, 8vo. pp. 55.

37. The British Medical Almanac for 1836; with Supplement. To be continued annually. London, 1836, 12mo. pp. 160.

38. Rudiments of Physiology. In three Parts. By John Fletcher, M.D., &c. Edin. 1835, 8vo.

39. Syllabus of Lectures on the Diseases of the Nervous System; to be delivered in the Aldersgate School of Medicine. By M. Hall, M.D. &c. Lond. 1835. 8vo. pp. 19.

40. An Introductory Address, delivered at the Commencement of the Tenth Session of the Manchester School of Medicine and Surgery, Pine Street. By James L. Bardsley, M.D., &c.—London, 1835. 8vo. pp. 47.

41. An Address delivered at the First Anniversary Meeting of the Birmingham School of Medicine and Surgery. By James Thomas Law, Chancellor of the Diocess of Litchfield. Birmingham, 1835. 8vo.

42. A Treatise on the Functional and Structural Changes of the Liver in the process of Disease; and on the agency of Hepatic Derangements in producing other Disorders. By W. E. E. Conwell, Surgeon, London, 1835, 8vo. 14s.

43. Clinical Illustrations of the more important Diseases of Bengal, with the result of an Inquiry into their Pathology and Treatment. By William Twining, First Assistant Surgeon, General Hospital, Calcutta. Calcutta, 1835. Second Edition, 2 vols. 8vo. pp. 481, 438. 24s.

44. Supplement to Outlines of Physiology. By W. P. Alison, M.D., F.R.S.E. Professor of the Institutes of Medicine in the University of Edinburgh. Edin. 1836.

45. Manual of Practical Midwifery; containing a description of natural and difficult Labours, with their Management. Illustrated by 15 Engravings. By James Reid, M.D. Surgeon to the Parochial Infirmary of St. Giles, &c. London, 1836, 12mo. pp. 246. 5s. 6d.

46. Tabula Nosologiæ et Historiæ Morborum. By J. R. Nichols, Lond. 1835.

47. A Lecture introductory to the Study of Medical Science, delivered at the Opening of the Andersonian University, Session 1835–6. By Robert Hunter, M.D. Glasgow, 1835. 8vo. pp. 32.

48. A new and complete Manual of Auscultation and Percussion, as applied to the Diagnosis of Diseases. By M. A. Racilorski, M.D. of the Faculty of Paris, translated by William Fitzherbert, B.A. Cambridge, 1 vol. 12mo. 5s.

